# Manufacture of low thermal conductivity cordierite ceramic foam based on low-cost waste and raw materials

**DOI:** 10.1038/s41598-025-09747-9

**Published:** 2025-07-12

**Authors:** Esmat M. A. Hamzawy, Bassem S. Nabawy, Gehan T. El-Bassyouni, Zeinab A. Abd El-Shakour

**Affiliations:** 1 Glass Research Department, National Research Centre, Cairo, Egypt; 2 Geophysical Sciences Department, National Research Centre, Cairo, Egypt; 3 Refractories, Ceramics, and Building Materials Department, National Research Centre, Cairo, Egypt; 4 Geological Sciences Department, National Research Centre, Cairo, Egypt

**Keywords:** Ceramic foam, Cordierite, Thermal insulator, Magnesite, Silica fumes, Aluminum slag, Ceramics, Sustainability

## Abstract

Magnesite, silica fumes, and aluminum slag were used to create foam cordierite ceramic material. During the sintering process, cordierite ceramic was formed at temperatures between 1200° and 1350°C. Between 1250° and 1300°C during the sintering process, the samples experienced swelling and an increase in porosity. However, the existence of holes and tiny openings in the samples indicates an alteration in their overall microstructure. Scanning electron microscopy (SEM) reveals that the pore size increases at elevated sintering temperatures, particularly at 1300°C. The submicron and nanoscale particles are dispersed within a glassy matrix. The apparent density and specific gravity values range from 0.498 to 1.501 g/cm^3^ and from 1.677 to 2.996 g/cm³, respectively. The measurements were conducted on foam samples molded at 20 and 30 kN, revealing a little rise in the apparent density and a decrease in porosity with the elevation of molding pressure. The apparent density (ρ_b_) and the porosity values (∅_He_, 48.22–83.16%.) are directly and inversely proportional correlations to the thermal conductivity (R² = 0.982 & R² ≥ 0.963, respectively). Furthermore, the thermal conductivity is controlled positively by the thermal diffusivity (1.192 ≤ ∝ ≤ 1.882 mm²/s, R² ≥ 0.848) and heat capacitance (604.9 ≤ Cp ≤ 841.4 J/(K·kg), R² ≥ 0.946). This lightweight ceramic foam, once sintered at 1300°C, serves as an effective heat insulator, which appears to be the optimal temperature for achieving superior thermal properties in insulating cordierite foam.

## Introduction

Cordierite (Mg₂Al₄Si₅O₁₈) is an important ceramic in the MgO-Al_2_O_3_-SiO_2_ system, and it was designed to be a good candidate for various industrial applications, including thermal insulation, biological or catalytic substrate, gas adsorption, mass separation, and water purification^1– 2^. Cordierite ceramics are synthesized using many processes, including crystallization from glasses, single crystal growth, a sol-gel approach, and combustion synthesis^3^. The cordierite phase can be divided into three polymorphic systems based on temperature: (a) low temperature phase (µ-cordierite), (b) low temperature phase (β-cordierite), and (c) high temperature phase (α-cordierite)^4^.

Cordierite ceramic is significant due to its unique properties, such as low dielectric constant and thermal expansion coefficient (CTE)^5^, high resistance to thermal shock, high temperature properties, and good surface and mechanical properties^6^. Low CTE materials are important for various applications, including household cookware, supersonic aircraft glass, turbine engine heat exchangers, and high-accuracy optical movements^7^. Furthermore, their low permittivity and high resistivity of cordierite ceramic allow for a wide range of applications^8^.

Simple techniques for making cordierite ceramics involve heating different silicon oxides, aluminum, and magnesium that have the same chemical makeup as cordierite, as well as using natural materials like talc and kaolinite that contain SiO₂, Al₂O₃, and MgO^9– 10^. As a result, to ensure the cordierite ceramics are free of impurities, careful attention was given during the sintering process based on the raw materials used in making cordierite^11^. Cordierite begins to develop noble properties as the predominant phase at 1350^o^C, associated with increasing the spinel content, as established by Araujo et al.^6^. They claimed that cordierite sintered through a diffusion process between MgO, Al_2_O_3_, and SiO_2_, rather than between silica and spinel. Nonetheless, increasing the sintering temperature from up to 1450^o^C reduced the spinel content and resulted in a complete vanishing of the cristobalite content, resulting in a growth in the standard formulation of the cordierite content to nearly 81 wt%, indicating that the cordierite formation is mostly due to interdiffusion between cristobalite and spinel^12– 13^. As a result, it is possible that increasing the sintering temperature will cause an increase in density and a decrease in porosity. The cordierite ceramic has a limited range of sintering temperature, and sintering is not easy without using a sintering additive to initiate the liquid phase formulation^14– 15^.

Silica fume is a byproduct of the reduction of SiO_2_ with carbon in the presence of iron at a temperature of 1750°C. Silica fume is derived from the commerce of ferro-silicon alloys and silicon metal. It consists of tiny particles with a significant surface area in the SiO_2_ range of 95–97 wt%. It could be utilized to enhance the characteristics of concrete^16^. Furthermore, silica fume has been shown to improve bond strength, abrasion resistance, and concrete compressive strength while also protecting steel from corrosion^17^. Aluminum is the most abundant element in the earth’s crust (8.2% by mass), after oxygen (46% by mass) and silicon (27.7% by mass). Aluminum slag is a byproduct of the aluminum melting process. It is more likely to be granular, comparable to sand; however, it contains an extremely high concentration of metal as well as lower levels of oxides and salts^18^.

**Fig. 1:**
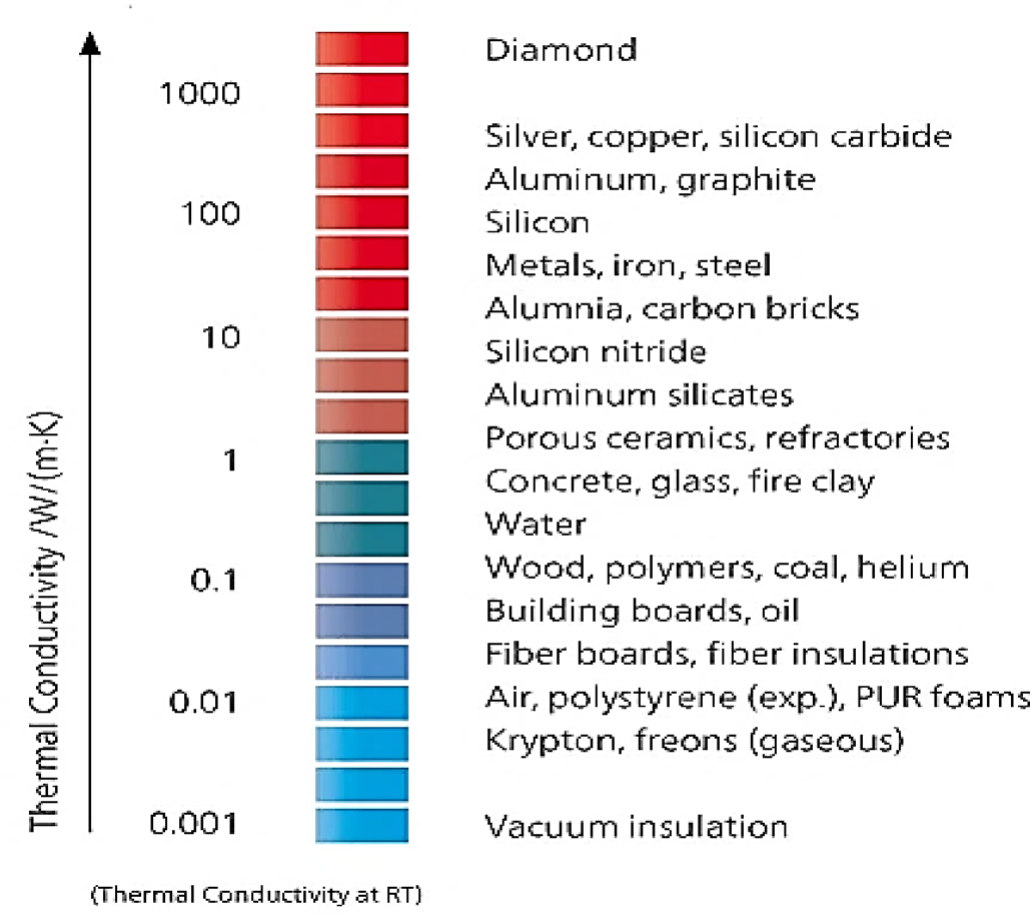
Thermal conductivity values of some selected materials at the room temperature (RT)^3,24^.

The thermal properties of materials, such as cordierite ceramic foam, are mainly influenced by their porosity and apparent density, which are key factors in their thermal properties like effusivity (ϒ), diffusivity (α), thermal conductivity (κ), and heat capacity (Cp), as explained in different literatures that talk about how the material can move, conduct, and hold heat^3^. The mineral composition and its spatial distribution, pore fluid saturation, porosity, pressure, and temperature also influence the thermal properties of solid materials^19– 20^. It ranges from 0.68 to 29.3 W/(m·K) for porous ceramics (e.g., cordierite ceramic foam), fired clays, glass, alumina, and concrete^21–23^ (Fig. 1). The study estimates the porosity, apparent density, and thermal properties of prepared cordierite ceramic foam samples to find out what properties they have and how well they might work as heat insulators?

In the current study, cordierite ceramic was prepared using Silica fume, Al-slag, and magnesite as starting materials. The scanning electron microscope (SEM) and X-ray diffraction (XRD) were utilized for characterizing the sintered samples. To verify the application of cordierite ceramic foam as a thermal insulator, some similar researches have been applied to ceramic foams manufactured using waste materials, e.g., anorthite, fly ash, and gypsum^3,25^.

## Experimental techniques

The design of the composition of the prepared samples was based on the stoichiometric cordierite (Mg₂Al₄Si₅O₁₈). The starting materials were Al-slag as the source of Al and silica fume as the source of silicon, while magnesite was the source of Magnesium. Table 1 presents the chemical composition of the used raw materials (silica fume, Al-slag, and magnesite), of the cordierite, and the of prepared batch.

Before the sintering process, the weighed cordierite sample has been well mixed with the starting materials in a ball mill for 4 h (Retsch GMBH west Germany Type S1 with a 1:1 batch-to-ball ratio at 300 rpm speed of revolution) to achieve a suitable micro grain size (nearly = 83 μm) and ensure the homogenization. Samples were then shaped into discs with a diameter of one inch using a uniaxial compressor (Paul Weber Matchinen Apparateebau-Germany) with 30 kN, using polyvinyl alcohol (7% PVA) as a binder material. For testing the implication of molding pressure, an additional group of samples was molded at 20 kN. The shaped samples were sintered at 550°C and then up to temperatures varying between 1200° and 1350°C (with a heating rate of 10 °C/min for all heating routes).

Identification of the crystalline phases after sintering was considered using an X-ray diffractometer (model BRUKER Axs, D8 ADVANCE) and the Match program. The microstructures of the sintered samples were examined using SEM imaging (SEM/EDX SEM Model Quanta 250), which captured images of the surface after it was treated with a solution of 1% HF and 1% HNO₃.

**Table 1:** Chemical composition (in wt%) of the starting materials, the cordierite, and the prepared batch.

Materials	Chemical composition of the starting materials															
	SiO_2_	Al_2_O_3_		Fe_2_O_3_	CaO		MgO	Na_2_O	K_2_O	MnO		TiO_2_	Cl		SO_3_	I.L
Al-slag	5.66	88.94		1.75	1.25		1.0	0.2	0.1	0.1		0.19	0.01		0.3	0.5
Silica Fume	94.96	0.97		0.96	0.75		0.45	0.28	0.25	0.02		0.01	0.02		0.34	0.99
Magnesite	0.80	0.05		0.74	3.36		43.94	nd	nd	nd		nd	nd		nd	51.11

Cordierite (Mg₂Al₄Si₅O₁₈) composition ( Wt.%)			Batch composition in grams		
MgO	Al_2_O_3_	SiO_2_	Magnesite	Al-Slag	Silica Fume
13.78	34.86	51.36	31.36	39.19	54.27

The dry weight (wt) of the discs was determined using a digital balance with an accuracy of 0.1 mg, while their dimensions and bulk volumes (vb) were assessed with a digital caliper that has an accuracy of 0.01 mm. The grains’ volume (vg) has been measured using a helium pycnometer (UltraPyc 1200e of Quantachrome) at a pressure of 15 psi (0.1034 MPa) and a temperature of 20°C. The apparent density (ρ_b_ in g/ cm^3^), the specific gravity (ρ_SP_ in g/ cm^3^), and porosity (∅_He_) have been subsequently calculated utilizing the equations and mathematical models that were introduced by Nabawy^26– 27^.

1 $$\emptyset _{{He}} = {\text{ }}100{\text{ }}x{\text{ }}\left( {\rho _{{SP}} - \rho _{b} } \right)/\rho _{{SP}}$$

2 $$\rho _{{SP}} = {\text{ }}wt/vg,~~~~~~~\rho _{b} = {\text{ }}wt/vb$$

To get accurate, high-precision measurements, the density and porosity assessments were conducted five times for each sample, with the average result regarded as the characteristic measurement. The standard deviation was calculated for each sample. The measurement protocol has been extensively detailed by several authors^28–32^. The heat flow in a matter is governed by the heat gradient (G); hence, the thermal conductivity (κ) is calculated utilizing the subsequent model.

3 $$\kappa = {\text{ }}\left( {{\text{Q}}/{\text{G}}} \right)*\left( {{\text{L}}/{\text{A}}} \right)$$

where Q is the amount of heat flow perpendicular to its surface, at a heat gradient G through a sample of dimensions A and L, and κ is the thermal conductivity (in W/(m.K)). In addition, κ is dependent on density (ρ in kg/m^3^), heat capacity (Cp in J/(kg·K)), and thermal diffusivity (α in m^2^/s). Therefore, we can calculate the thermal diffusivity and effusivity as follows:

4 $$\alpha {\text{ }} = \kappa/\left( {\rho .{\text{Cp}}} \right)$$

5 $$\Upsilon = {\text{ }}(\kappa.\rho .Cp)^{{1/2}} = \kappa/\alpha ^{{1/2}}$$

We tested the thermal properties of the synthesized cordierite ceramic foam samples using the Transient Hot Bridge method. We employed the method to determine the heat capacity, thermal effusivity, diffusivity, and thermal conductivity for each sintering phase. The main benefit of this method is that it lets you measure both thermal conductivity and diffusivity at the same time, and it also takes a lot less time to do than traditional methods. The concept involves placing a strip-shaped thermal conductor between two sets of highly smooth parallel surfaces. This conductor functions as a temperature sensor and a source of heat simultaneously. Each sample pair is subjected to a specific heat flow from the sensor, resulting in a temperature increase that facilitates the measure of the different thermal parameters. Some literature described and explained the used idea and method and a thorough look at the different thermal parameters^19,33^.

## Results

### Phases characterization using XRD

**Fig. 2:**
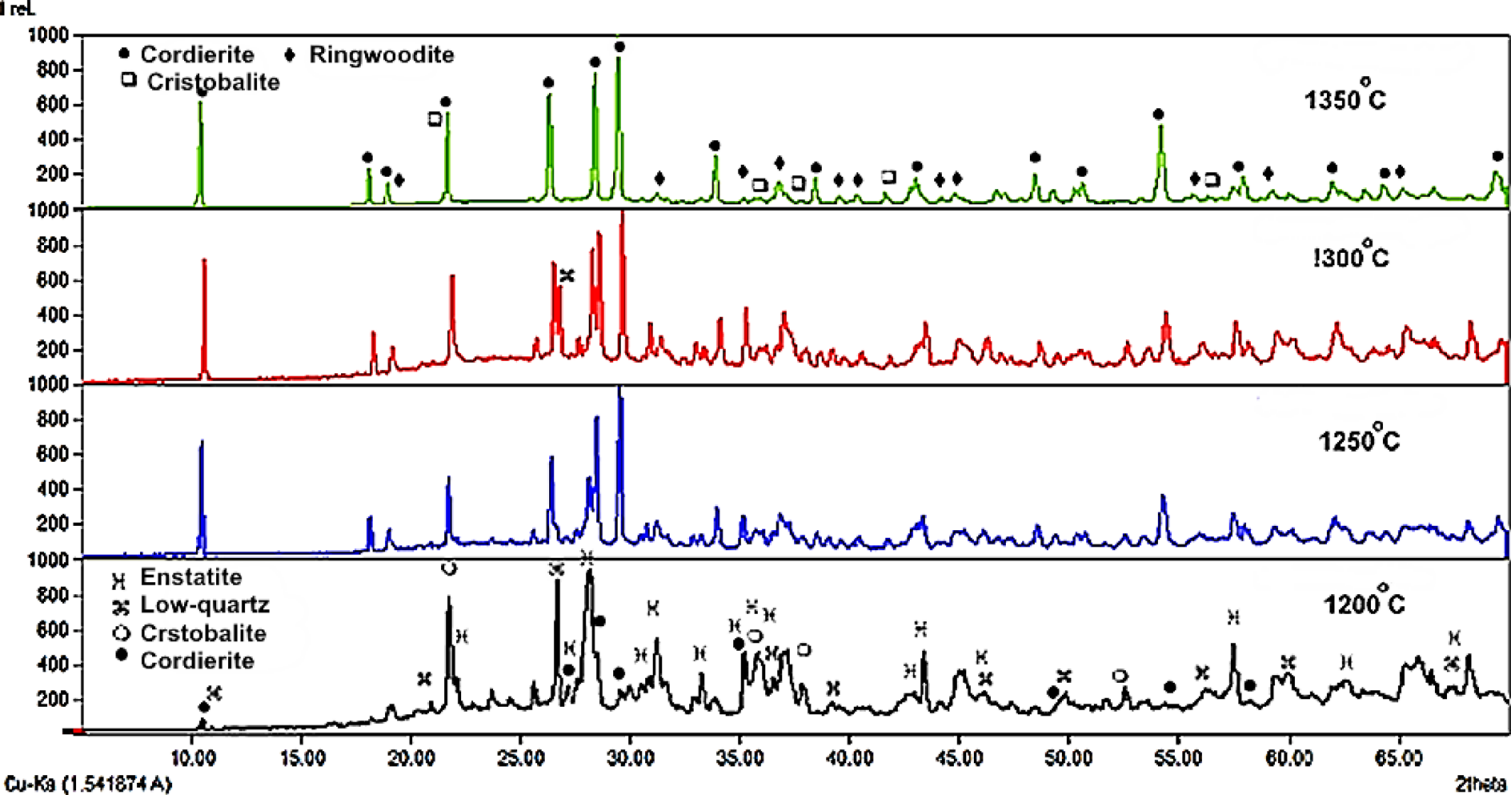
X-ray diffraction patterns of disc-shaped samples sintered in between 1200° and 1350°C/2 h.

The prepared stoichiometric disc-shaped samples were sintered incrementally for two hours in the range of 1200°C and 1350°C with increasing temperature equal to 50°C/2 hours. According to the XRD data, the sample sintered at 1200°C shows the crystallization of enstatite (56.6%, (Mg_1.66_Al_0.4_Ar_0.14_Fe_0_.24)Si_1.94_O_6_, ICDD: 96-900-6440), low-quartz (17.1%, SiO_2_, ICDD: 96-900-0145), and cristobalite (8.9%, SiO_2_, 96-900-0145) with little cordierite (17.4%, (Mg_1.91_Fe_0.09_)Al_4_Si_5_O_18_, ICDD: 96-900-9688) (Fig. 2). However, at higher temperatures between 1250° and 1350 °C/2 h, cordierite became the major phase with little ringwoodite (Mg_1.94_SiO_4_, ICDD: 96-901-4472) and cristobalite (Fig. 2).

**Fig. 3:**
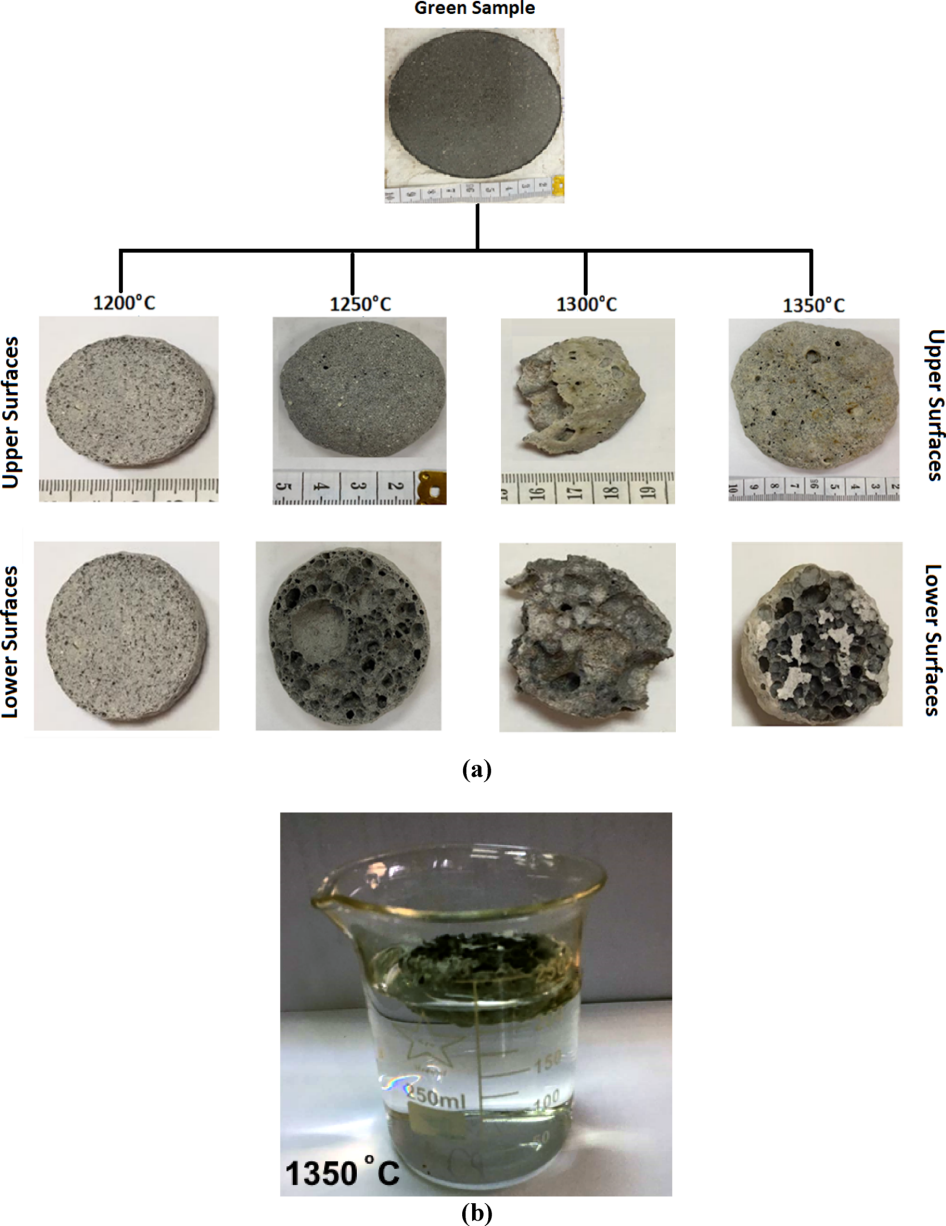
(**a**) The sintered disc-shaped cordierite samples at various temperatures (1200°, 1250°, 1300°, and 1350° (C) float in water, and (**b**) the foam nature of the sintered samples floating on the water surface.

Rehabilitated highly vesicular, and vuggy nature of the sintered samples can be easily noticed and examined on the mega scale, where surface swelling is shown in the various samples with more vesicular pores at higher sintering temperatures (Fig. 3a). The sample sintered at 1200°C is relatively dense, but samples sintered at temperatures above 1200°C show swelling due to self-foaming of their constituents and expansion of their materials, creating different sizes of pores. This swelling is possibly because of the oxygen being released from the magnesite or changing the bonding^34–36^. The three sintered samples at 1250°, 1300°, and 1350°C float on the surface of the distilled water due to the decrease of the samples’ density to less than the density (< 1.00 g/cm^3^) (Fig. 3b).

### Microstructure characterization based on SEM imaging

**Fig. 4:**
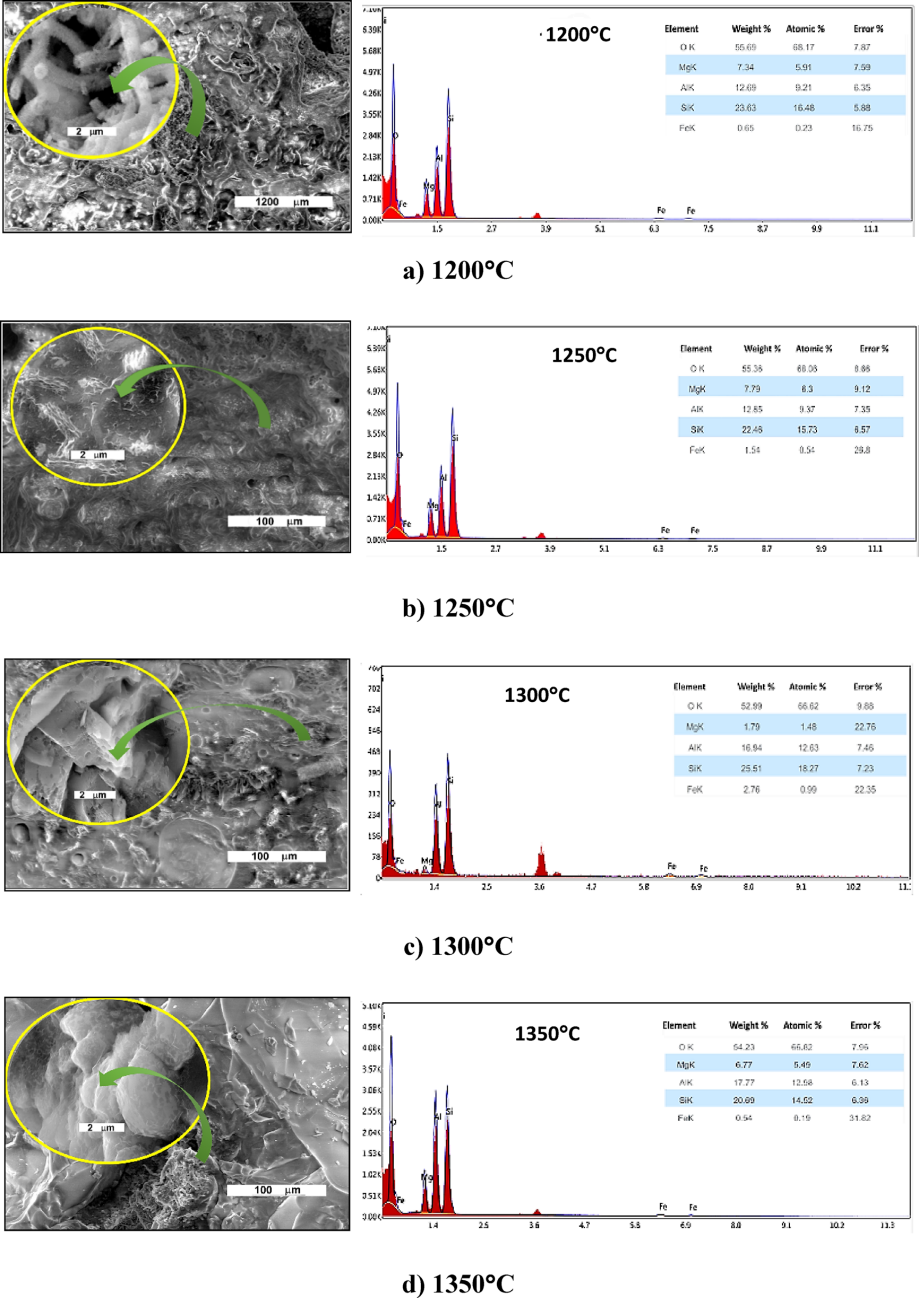
SEM photographs and EDX microanalysis of the sintered samples at 1200°, 1250°, 1300°, and 1350°C

The SEM examination of the sintered samples at 1200°, 1250°, 1300°, and 1350 °C shows different microstructures (Fig. 4). At 1200°C the specimen shows fibrous-like spread through pores, while at 1250°C threadlike crystals in nano size spread in cryptocrystalline groundmass^37^. At 1300°C, massive irregular surfaces show fine needle crystals, and accumulated eu- and subhedral crystals spread in cryptocrystalline groundmass also at 1350°C/2 h was similar to the later one (Fig. 4). The X-ray microanalysis of the samples reveals that they contain cordierite .

### Physical characterization of the sintered cordierite samples

The measured physical properties of the molded cordierite discs at 20 kN declare that the specific gravity and the apparent density range from 2.996 to 2.700 g/cm^3^ and from 0.498 to 1.471 g/cm^3^, respectively. These values are coincident with increasing the sintering temperatures from 1200° to 1350 °C with very high reliability (St. Dv. ≤ 0.0021, Table 2). The measured ∅_He_ varies from 53.16% (at 1200°C) to 83.16% (at 1350°C). The thermal capacitance (Cp), diffusivity (α), effusivity (ϒ), and thermal conductivity (κ) are in the range of 629.1–841.4 J/(K.kg), 1.201–1.853 mm^2^/s, 578.6–993.8 W(s)^0.5^/m^2^K, and 0.4097–2.2939 W/(m.K), respectively **(**Table 2**).** The lowest thermal capacitance, diffusivity, effusivity, and thermal conductivity are mostly assigned to the samples sintered in the range of 1300°-1350°C.

Applying more molding pressure (30 kN) to another group of samples (group II), sintered at the same conditions as group I, shows that the apparent density and specific gravity values vary in the range of 0.507–1.501 g/cm^3^ and 1.677–2.840 g/cm^3^, respectively. The estimated helium porosity varies from 48.22% (1200°C) to 70.31% (at 1350°C) (Table 2). Therefore, increasing the molding pressure slightly increased the apparent density and decreased the porosity, i.e., increased the created pore volume.

**Table 2:** The thermal properties, apparent density, specific gravity, and porosity of the molded cordierite foams.

Group	Sample No.	Temp.	∅_He_ %	ρ_b_ g/cm^3^	ρ_SP_ g/cm^3^	St. dv.	κ W/(m.K)	α mm^2^/s	Cp J/(K.kg)	ϒ W(s)^1/2^/m^2^K
Group I, 20 kN	1	1200 °C	53.16	1.471	2.996	0.0005	2.2939	1.853	841.4	993.8
	2	1250 °C	76.14	0.587	2.702	0.0021	0.4434	1.201	629.1	975.3
	3	1300 °C	83.16	0.498	2.700	0.0005	0.4097	1.302	632.1	657.1
	4	1350 °C	66.03	0.958	2.759	0.0019	1.0532	1.675	656.2	578.6
Group II, 30kN	5	1200 °C	48.22	1.501	2.841	0.0067	2.2208	1.882	802	740.7
	6	1250 °C	68.62	0.591	1.869	0.0632	0.4231	1.192	604.9	987.9
	7	1300 °C	70.31	0.507	1.677	0.0725	0.4548	1.394	655.2	656.7
	8	1350 °C	58.81	0.985	2.325	0.0963	1.0799	1.616	697.7	568.4

Temp. is the sintering temperature, ∅_He_ is the porosity, ρ_b_ and ρ_SP_ are the apparent density and specific gravity, respectively, St.dv. is the standard deviation of the ρ_SP_, ϒ is the thermal effusivity, Cp is the specific heat, α is the thermal diffusivity, and κ is the thermal conductivity.

## Discussion

### Impacts of the foam nature on density and porosity

**Fig. 5:**
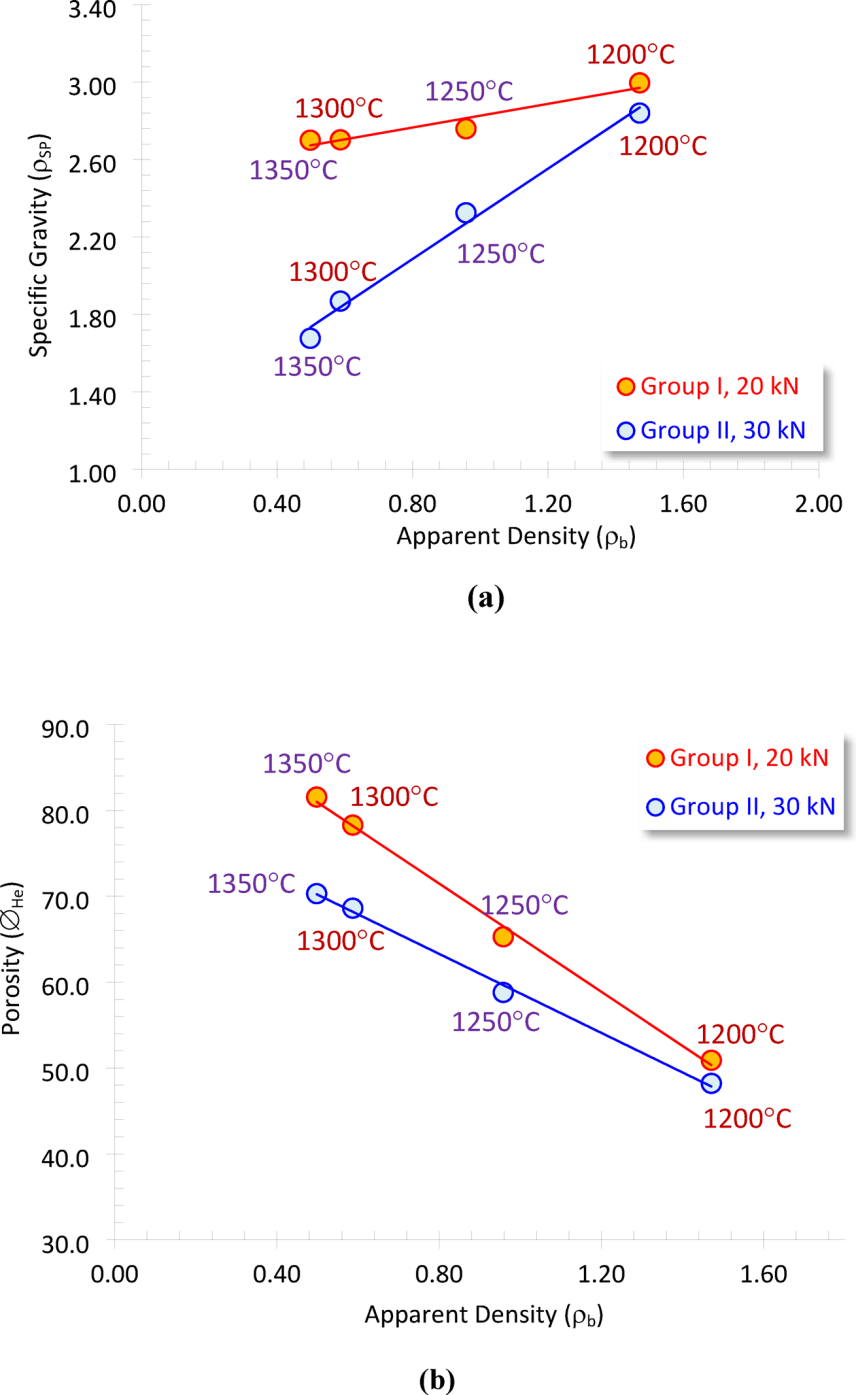
Plotting the apparent density versus (**a**) specific gravity, and (**b**) porosity of the sintered samples for group I (molded at 20kN), and Group II (molded at 30 kN).

Plotting the specific gravity versus the apparent density of the prepared cordierite samples indicates a highly reliable direct proportional relationship (R^2^ ≥ 0.926, Fig. 5a). Although the apparent density is dependent on both the specific gravity and porosity, its highly dependance on the specific gravity is not theoretically assigned^38,39^. Furthermore, changing the specific gravity with the sintering temperature may refer to the creation of some isolated vesicles within the foam matrix, which are estimated as a part of the grains’ volume, giving rise to less specific gravity values, i.e., the measured specific gravity is pseudo due to the dominance of the isolated vesicles (Fig. 3a, b).

Additionally, presenting the porosity as a function of the density is a common plot, where improving porosity from 0%, which corresponds to the specific gravity^3,40^. As a result, showing the porosity of the molded cordierite foam samples in relation to their apparent density reveals a reverse relationship, with very high accuracy (R² ≥ 0.996, Fig. 5b), which means that we can use this mathematical equation to calculate the porosity of the foams based on their apparent density^41–43^.

Both the specific gravity and ∅_He_ values can be calculated for the various sintered foams in terms of their apparent density, considering the molding pressure as follows.

Group I, 20 kN:

6 $$\rho _{{SP}} = {\text{ }}2.52{\text{ }} + {\text{ }}0.306\rho _{b} ~~~~~~~\left( {R^{2} {\text{ }} = {\text{ }}0.926} \right)$$

7 $$\emptyset _{{He}} = {\text{ }}96.69{\text{ }} - 31.52\rho _{b} ~~~~~~~~~~~~\left( {R^{2} {\text{ }} = {\text{ }}0.996} \right)$$

Group II, 30 kN:

8 $$\rho _{{SP}} = {\text{ }}1.15{\text{ }} + {\text{ }}1.166\rho _{b} ~~~\,\,\,\left( {R^{2} {\text{ }} = {\text{ }}0.990} \right)$$

9 $$\emptyset _{{He}} = {\text{ }}81.70{\text{ }} - 23.0\rho _{b} ~~~~~\,\,\left( {R^{2} {\text{ }} = {\text{ }}0.997} \right)$$

### Thermal conductivity

#### Implication of the thermal conductivity on the physical parameters of the cordierite

**Fig. 6:**
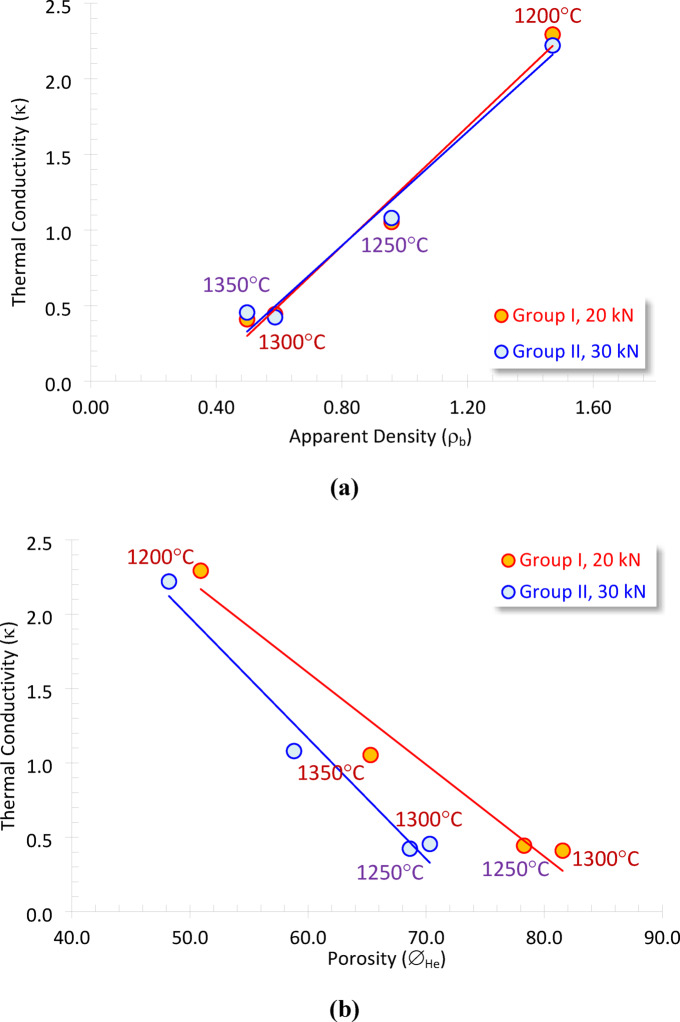
Plotting the thermal conductivity (κ) as a function of (**a**) apparent density (ρ_b_), and (**b**) porosity (∅_He_) of the various sintered samples at the two molding pressures.

Producing ceramic foam with low κ necessitates augmenting the volume of the discrete and interconnected vesicles constituting the material’s porosity, hence reducing its apparent density. Consequently, both the apparent density and porosity predominantly influence thermal conductivity^20,23,25^. We determined the heat absorption capacity of cordierite ceramic foam at room temperature and subsequently correlated this value with its ρ_b_ and ∅_He_ (Fig. 6a, b). This graph illustrates that κ of the prepared samples is influenced by both the apparent density (ρ_b_) and the porosity (∅_He_). The direct and inverse proportional connections exhibit a robust correlation (R² ≥ 0.963). The correlations identified in the multiple regression analysis are highly reliable, allowing us to derive a positive estimation of κ regarding both the ρ_b_ and the ∅_He_ as follows.

Group I, 20 kN:

10 $$ \kappa = {\text{ }}1.97\rho _{b} - {\text{ }}0.68~~~~~~~~~\left( {R^{2} {\text{ }} = {\text{ }}0.982} \right)$$

11 $$ \kappa = {\text{ }}5.32{\text{ }} - {\text{ }}0.062\emptyset _{{He}} ~~~~~~~~\left( {R^{2} {\text{ }} = {\text{ }}0.963} \right)$$

Group II, 30 kN:

12 $$\kappa = {\text{ }}1.88\rho _{b} - {\text{ }}0.61~~~~~~~~~~~~~~~~\left( {R^{2} {\text{ }} = {\text{ }}0.982} \right)$$

13 $$ \kappa = {\text{ }}6.04{\text{ }} - {\text{ }}0.081\emptyset _{{He}} ~~~~~~~~~~~~~~~~~~\left( {R^{2} {\text{ }} = {\text{ }}0.971} \right)$$

From this plot, it can be stated that the molding pressure has minimal or no effect on the thermal conductivity values, as the slight increase in molding pressure does not significantly influence the physical properties of the prepared cordierite. A minor elevation in thermal conductivity is seen at elevated temperatures (1300°-1350°C, Table 2; Fig. 6a, b).

The significant reliance of thermal conductivity on both ρ_b_ and ∅_He_ can be elucidated by the composition of solid materials, which consist of two complementary phases: the solid phase, which can function as a thermal conductor or insulator, and the pore phase, which serves as a thermal insulator. This relationship accounts for the inverse proportionality between thermal conductivity and porosity^44– 45^.

Estimating the thermal conductivity of the solid and saturating-pore fluids indicates that the thermal conductivity of foams slightly increases with increasing the foam density, which can be attributed to interfacial phonon scattering^46^.

A lot of research has been done on the connection between thermal conductivity and porosity^20,22,25^, but there is still no one model that can be used to predict thermal conductivity based on density or porosity. Understanding this requires considering factors beyond porosity and density. It is also affected by the pore types, sizes, and connectivity, and their spatial distribution. It is also affected by the solid phase’s texture and structure, as well as its main parts. We must not disregard the characteristics of the pore, such as its size and dispersion. However, some literature has presented alternative equations, particularly those proposed by Nabawy and Géraud^22^ and Hamzawy et al.^3^.

Nabawy and Géraud^22^:

14 $$\kappa = {\text{ }}1.26\rho _{b} - {\text{ }}0.54~~~~~~~~\left( {R^{2} {\text{ }} = {\text{ }}0.518} \right)$$

15 $$\kappa = {\text{ }}3.82{\text{ }} - {\text{ }}0.058\emptyset _{{He}} ~~~~~~~~~~~~~\left( {R^{2} {\text{ }} = {\text{ }}0.852} \right)$$

Hamzawy et al.^3^:

16 $$\kappa = {\text{ }}0.974\rho _{b} + {\text{ }}0.24~~~~~~~~~~~~~~~~\left( {R^{2} {\text{ }} = {\text{ }}0.966} \right)$$

17 $$ \kappa = {\text{ }}3.21{\text{ }} - {\text{ }}0.031\emptyset _{{He}} ~~~~~~~~~~~~~~~~~~~~\left( {R^{2} {\text{ }} = {\text{ }}0.954} \right)$$

A comparison of the models by Nabawy and Géraud^22^ and Hamzawy et al.^3^ with the models presented in this study reveals comparable multiplication factors; however, the current models exhibit greater reliability (R² = 0.963–0.982) than those proposed by the aforementioned authors. The reliability of these models may stem from their reliance on synthetic materials, primarily composed of anorthite. This approach differs from the models proposed by Nabawy and Géraud^22^, which are predicated on actual sandstone rocks, wherein the pores may lack connectivity or uniform distribution.

Despite extensive study on the relationship between thermal conductivity and porosity^20,22,25^, no singular universal model exists for predicting thermal conductivity based on density or porosity. This can be elucidated by the observation that κ is influenced not only by porosity and density but also by supplementary governing elements, such as pore type, size, connectivity, and spatial distribution, as well as the structure and texture of the solid phase and its principal components. The characteristics of the pore, including pore size and dispersion, must not be disregarded. However, some literature has presented alternative Eqs.^3,22^.

Furthermore, a study of the thermal conductivity of synthetic cordierite foams (κ = 0.4097 W/(m.K) at ∅_He_ = 83.16%, temperature 1300°C, and 20 kN molding pressure) was compared to that of anorthite ceramic foam made from fly ash and gypsum (κ = 0.042 W/(k·m) at ∅_He_ = 94%)^25^ and that made from silica fume, aluminum slag, and limestone (κ = 0.8086 W/(m.K) at ∅_He_ = 81.56%) by Hamzawy et al.^3^ and Su et al.^47^.

Higher porosity values are needed to get lower thermal conductivity values. We used the derived ρ_b_-∅_He_ model from Eqs. (11 and 13) to figure out how well the synthetic cordierite ceramic foam made of magnesite, silica fume, and aluminum slag would conduct heat. At this porosity, the anticipated thermal conductivity approaches negligible values near zero. This ceramic foam doesn’t conduct heat very well, about the same as polypropylene (κ = 0.22 W/(m.K)), low-density polyethylene (κ = 0.33 W/(m.K)), silicon, polycarbonate, insulating bricks (κ = 0.15–0.47 W/(m.K)), and crystalline sulfur (κ = 0.20 W/(m.K))^49– 50^. This could be also explained by the fact that the relatively low thermal conductivity of Phase-Change Material (PCM) reduces the efficiency of the material to storage heat^51^.

#### Impacts of thermal conductivity on Cp and α

**Fig. 7:**
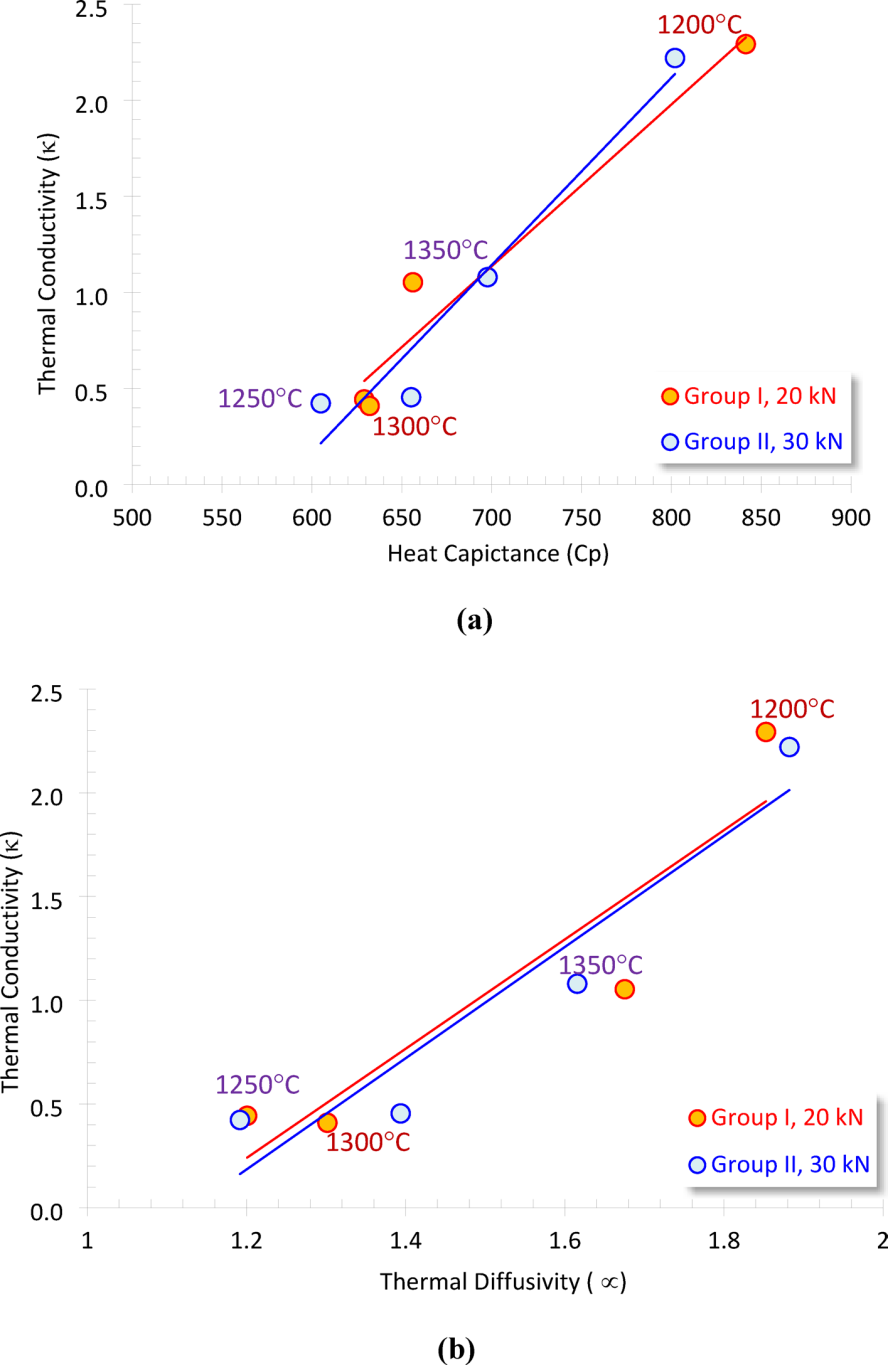
Plotting the thermal conductivity (κ) as a function of (**a**) heat capacitance (Cp) and (**b**) thermal diffusivity (α) of the various sintered samples at the two molding pressures.

The thermal conductivity (κ) is fundamentally dependent on the apparent density, thermal diffusivity (α), and heat capacity (Cp) (Eqs. 4 & 5). In other words, Consequently, plotting k against α and Cp is a recognized method for identifying its primary controlling factors (Fig. 7a, b). κ is primarily governed by the specific heat capacity (Cp), which reflects the material’s heat storage capacity, and by the thermal diffusivity, which indicates the rate of heat transfer through the material. In other words, thermal conductivity refers to the ability of a material to conduct heat, while the heat capacity indicates how much heat energy is released or absorbed^52– 53^.

The thermal conductivity is directly proportional to both thermal diffusivity and heat capacity, demonstrating a high reliability (R² = 0.848–0.950). Consequently, thermal conductivity (κ) can be estimated as a function of these thermal parameters using the following mathematical models.

Group I:

18 $$\it \kappa = {\text{0.0084 Cp - 4}}{\text{.759}}  \left( {{\text{R}}^{{\text{2}}} {\text{ = 0}}{\text{.950}}} \right)$$

19 $$\kappa = {\text{ }}2.634 \alpha - {\text{ }}2.921~~~~~~~~~~~~\left( {R^{2} {\text{ }} = {\text{ }}0.848} \right)$$

Group II:

20 $$\kappa = {\text{ }}0.0098{\text{ }}Cp{\text{ }}{-}{\text{ }}5.686~~~~~~~~~~~~~\left( {R^{2} {\text{ }} = {\text{ }}0.946} \right)$$

21 $$\kappa = {\text{ }}2.680\alpha - {\text{ }}3.032~~~~~~~~~~~~~~~~~\left( {R^{2} {\text{ }} = {\text{ }}0.846} \right)$$

Nabawy^20^ published two mathematical models concerning κ to α and Cp in his study of a specific meteorite sample (natural material), demonstrating a relatively high level of reliability.

This study compares the two models proposed by Nabawy^20^ with the existing models for the prepared anorthite ceramic foam. The existing models exhibit a comparatively elevated R² value. The synthetic nature of the prepared samples likely accounts for a controlled and well-mixed primary composition^20,54– 55^. Nonetheless, this comparison indicates that the multiplication factors of the κ-Cp model are approximately equal to 0.003 and 0.008, while the constant values of the κ-∝ equal to -1.792 and − 1.837 for the present study and that published by Nabawy^20^, respectively. Measuring the thermal parameters of additional samples with greater data availability may aid in the development of universal mathematical models.

## Conclusions

Solid waste and raw materials like magnesite, aluminum slag, and silica fume were used to make foamed ceramic that is made up of cordierite (Mg₂Al₄Si₅O₁₈). We synthesized cordierite, enstatite, low-quartz, and cristobalite by sintering an unaltered sample at temperatures ranging from 1200°C to 1350°C. The samples sintered at temperatures ranging from 1200°C to 1350°C exhibit a relationship between swelling and low density, particularly at 1300°C. As the temperature increases, the microstructure displays larger pores and vesicles. Furthermore, submicron and nanoscale particles are distributed throughout the glassy matrix, especially at temperatures ranging from 1250° to 1350°C. The apparent density and specific gravity values, when molded at 20 kN and 30 kN, vary from 0.498 to 1.501 g/cm³ and from 1.677 to 2.996 g/cm³, respectively. This number (0.4097 ≤ κ ≤ 2.2939 W/(m.K)) is related to the porosity in a way that is opposite to the apparent density. The porosity ranges from 48.22 to 83.16%. In addition, thermal conductivity is connected to both thermal diffusivity (1.192 ∝ ≤ 1.882 mm²/s) and heat capacitance (604.9 ≤ Cp ≤ 841.4 J/(K·kg)). Therefore, the main output of this study is manufacturing a lightweight ceramic foam capable of withstanding temperatures up to 1300 °C.

## Data Availability

Data will be available on reasonable request by contacting the corresponding author: Bassem S Nabawy; bsnabawy@yahoo.co.uk; bs.nabawy@nrc.sci.eg.

## References

[1] Chowdhury, A. et al. Synthesis, properties and applications of cordierite ceramics, part 2. *Interceram* **56** (2), 98–102 (2007).

[2] Fotoohi, B. & Blackburn, S. Effects of mechanochemical processing and doping of functional oxides on phase development in synthesis of cordierite. *J. Eur. Ceram. Soc.* **32**, 2267–2272 (2012).

[3] Hamzawy, E. M. A., El-Bassyouni, G. T., Abd El-Shakour, Z. A. & Nabawy, B. S. Manufacture of low thermal conductivity anorthite ceramic foam using silica fume, aluminum slag, and limestone. *Ceram. Int.* **51** (6), 7977–7985 (2025).

[4] Goren, R. & Ozgur, C. Gocmez, the Preparation of cordierite from talc, fly ash, fused silica and alumina mixtures. *Ceram. Int.* **32**, 53–56 (2006).

[5] Valášková, M. Clays, clay minerals and cordierite ceramics-A review. *Ceram. Silikáty*. **59**, 331–340 (2015).

[6] Araujo, P. A. S. et al. Cordierite-based ceramics with coffee husk Ash addition: I- microstructure and physical properties. *J. Mater. Res. Technol.* **15**, 2471–2483 (2021).

[7] García-Moreno, A. O., Fern´andezb, A. & Torrecillas, R. Sintering of multi –b-eucryptite ceramics with very low thermal expansion. *J. Mat. Res.* **103**, 4 (2012).

[8] Khattab, R. M., Sadek, H. E. H., Taha, M. A. & EL-Rafei, A. M. Recycling of silica fume waste in the manufacture of β-eucryptite ceramics. *Mater. Charact.* **171**, 110740 (2021).

[9] Hamzawy, E. M. A. & Ali, A. F. Sol-gel Preparation of Boron-containing cordierite Mg_2_(Al_4 – x_B_x_)Si_5_O_18_ and its crystallization. *Mater. Charact.* **57**, 414–418. 10.1016/j.matchar.2006.04.010 (2006).

[10] Alharbi, O. A. & Hamzaw, E. M. A. Sintered cordierite glass-ceramic bodies. *US Patent Application* 20120149542, Publication Date 14/6/2012.

[11] Ahmed, Y. M. Z., Ewais, E. M. M. & Amin, A. M. M. Processing of porous cordierite via the utilization of two waste metal oxides (Al-slag and silica fumes),* The 2012 World Congress on Advances in Civil, Environmental, and Materials Research (ACEM’ 12) Seoul, Korea* 26–30. (2012).

[12] Al-Harbi, O. A. & Hamzawy, E. M. A. Nanosized cordierite–sapphirine–spinel glass ceramics from natural Raw materials. *Ceram. Int.* **40** (4), 5283–5288 (2014).

[13] Njoya, D., Elimbi, A., Fouejio, D. & Hajjaji, M. Effects of two mixtures of kaolin-talc-bauxite and firing temperatures on the characteristics of cordierite- based ceramics. *J. Building Eng.* **8**, 99–106 (2016).

[14] Camerucci, M. A., Urretavizcaya, G. & Cavalieri, A. L. Sintering of cordierite based materials. *Ceram. Int.* **29**, 159–168 (2003).

[15] Hamzawy, E. M. A. & Al-Harbi, O. A. Sintered Mono-Cordierite Mg_2_Al4 – xBxSi_5_O_18_ Glass-Ceramic with B/Al Replacement at the Nano- and Micro-Scale. Silicon (10) 439–444. (2018). 10.1007/s12633-016-9471-3

[16] Hamzawy, E. M. A. & Binhussain, M. A. Sintered gahnite-cordierite glass-ceramic based on Raw materials with different fluorine sources. *Bull. Mater. Sci.* **38** (7), 1731–1736. 10.1007/s12034-015-1104-8 (2015).

[17] Pigeo, M. N., Plante, P. & Plante, M. Air void stability, part I: influence of silica fume and other parameters air void stability, part I: influence of silica fume and other parameters. *ACI Mater. J.* **86**, 482–490 (1989).

[18] Ali, M. M. & Yassen, R. S. Recovery of aluminum from industrial waste (Slag) by melting and electrorefining processes. *Al-Khwarizmi Eng. J.* **14** (3), 81–91 (2018).

[19] McKenna, T. E. Jr., Sharp, J. M. & Lynch, F. L. Thermal conductivity of Wilcox and Frio sandstones in South texas. Gulf of Mexico basin. *Bull. Am. Assoc. Pet. Geol.* **80** (8), 1203–1215 (1996).

[20] Nabawy, B. S. Frequency-based electric fingerprint and thermal properties of the NWA 869 chondrite. *J. Afr. Earth Sc.* **213**, 105232 (2024).

[21] Saiah, R., Perrin, B. & Rigal, L. Improvement of thermal properties of fired clays by introduction of vegetable matter. *J. Building Phys.* **34** (2), 124–142. 10.1177/1744259109360059 (2010).

[22] Nabawy, B. S. & Géraud, Y. Impacts of pore- and petro-fabrics, mineral composition and diagenetic history on the bulk thermal conductivity of sandstones. *J. Afr. Earth Sc.* **115**, 48–62 (2016).

[23] Rahmouni, A., Boulanouar, A., El Rhaffari, Y., Rezzouk, A. & Nabawy, B. S. Impacts of anisotropy coefficient and porosity on the thermal conductivity and P-wave velocity of calcarenites used as Building materials of historical monuments in Morocco. *J. Rock Mech. Geotech. Eng.* **15** (7), 1687–1699 (2023).

[24] WWW.Netzsch-thermal-analysis.com.

[25] Li, Y. et al. Fabrication and characterization of anorthite foam ceramics having low thermal conductivity. *J. Eur. Ceram. Soc.* **35** (1), 267–275 (2015).

[26] Nabawy, B. S. Impacts of dolomitization on the petrophysical properties of El-Halal formation, North sinai, Egypt. *Arab. J. Geosci.* **6** (2), 359–373 (2013).

[27] Nabawy, B. S. Impacts of fossil anisotropy on the electric and permeability anisotropy of highly fossiliferous limestone: a case study. *Mar. Geophys. Res.* **39** (4), 537–550 (2018).

[28] Nabawy, B. S., Sediek, K. N. & Nafee, K. N. S. A. Pore fabric assignment using electrical conductivity of some Albian–Cenomanian sequences in North Eastern desert, Egypt. *Arab. J. Geosci.* **8** (8), 5601–5615 (2015).

[29] Safa, M. G., Nabawy, B. S., Basal,., B. A. M. K., Omran, M. A. & Lashin, A. Implementation of a petrographical and petrophysical workflow protocol for studying the impact of heterogeneity on the rock typing and reservoir quality of reefal limestone: A case study on the Nullipore carbonates in the Gulf of Suez. *Acta Geol. Sinica*. **95** (5), 1746–1762 (2021).

[30] Hossein, H. A., Hamzawy, E. M. A., El-Bassyouni, G. T. & Nabawy, B. S. Mechanical and physical properties of synthetic sustainable geopolymer binders manufactured using rockwool, granulated slag, and silica fume. *Constr. Build. Mater.* **367**, 130143 (2023).

[31] Radwan, A. A., Nabawy, B. S., Kassem, A. A. & Elmahdy, M. An integrated workflow for seismic interpretation, petrophysical and petrographical characterization for the clastic Mangahewa reservoir in Pohokura gas field, Taranaki basin, new Zealand. *Geoenergy Sci. Eng.* **229**, 212117 (2023).

[32] Eysa, E. A., Nabawy, B. S., Ghoneimi, A. & Saleh, A. H. Petrophysical rock typing based on the digenetic effect of the different microfacies types of Abu Madi clastic reservoir in Faraskur gas field, onshore nile delta, Egypt. *J. Petroleum Explor. Prod. Technol.* **14** (2), 381–406 (2024).

[33] Maqsood, A. & Kamran, K. Thermophysical properties of porous sandstones: measurements and comparative study of some representative thermal conductivity models. *Int. J. Thermophys.* **26** (5), 1617–1632 (2005).

[34] Wang, S., Wang, H., Chen, Z., Ji, R. & Liu, L. Wang. Fabrication and characterization of porous cordierite ceramics prepared from fly Ash and natural minerals. *Ceram. Int.* **45** (15), 18306–18314 (2019).

[35] Barma, S. & Mandal, B. B., Effects of sintering temperature and initial compaction load on alpha-alumina membrane support quality.* Ceramics Int.*** 40** (7), Part B, 11299–11309, (2014).

[36] Kwon, S. T., Kim, D. Y. & Kang, T. K. Yoon, effect of sintering temperature on the densification of Al2O3. *J. Am. Soc. Ceram.* **70** (4), C–69 (1987).

[37] Eldera, S. S., Alharbi, O. A., Rüssel, C., Al-wafi, R. & Hamzawy, E. M. A. Nano-Scale cordierite Glass-Ceramic from natural Raw materials with different fluoride additions. *Silicon* **12** (5), 1051–1057. 10.1007/s12633-019-00200-x (2020).

[38] Jiang, Z., He, G., Shi, Y., Duan, Y., Lin, Y.,… Jiang, Y. (2024a). Contrasting effects of waste glass and scheelite tailings additions upon the properties of tailings-based foam ceramics and its mechanisms.* J. Cleaner Prod.*, 450, 142025.

[39] Jiang, Z., He, G., Duan, Y., Jiang, Y., Lin, Y., Zhu, Y.,… Wang, J. (2024b). Contrasting effects of various factors upon the properties of foam ceramics and the mechanisms of crystalline phase reconstruction and microstructure regulation.* Ceramics Int.* **50** (12), 21645–21657.

[40] Nabawy, B. S. & Wassif, N. A. Effect of the mineralogical composition on the petrophysical behavior of the amygdaloidal and vesicular basalt of Wadi wizr, Eastern desert, Egypt. *J. Afr. Earth Sc.* **134**, 613–625 (2017).

[41] Jiang, Z., He, G., Jiang, Y., Zhao, H., Duan, Y., Yuan, G.,… Fu, H. (2024). Synergistic preparation and properties of ceramic foams from wolframite tailings and high-borosilicate waste glass.* Construction and Building Materials*** 457**, 139367.

[42] De Carolis, S., Putignano, C., Soria, L. & Carbone, G. Effect of porosity and pore size distribution on elastic modulus of foams. *Int. J. Mech. Sci.* **261** (2024), 108661. 10.1016/j.ijmecsci.2023.108661 (2024).

[43] Apostolopoulou-Kalkavoura, V. et al. Effect of density, phonon scattering and nanoporosity on the thermal conductivity of anisotropic cellulose nanocrystal foams.* Sci. Rep.*** 11**, 18685 (2021). (2021). 10.1038/s41598-021-98048-y 10.1038/s41598-021-98048-y PMC8455657 34548539

[44] Ji, R., Liu, Y., Zhang, Y. & Wang, F. Machining performance of silicon carbide ceramic in end electric discharge milling. *Int. J. Refract. Met. Hard Mater.* **29** (1), 117–122. 10.1016/j.ijrmhm.2010.09.001 (2011).

[45] Ji, R., Liu, Y., Zhang, Y., Cai, B., Ma, J.,… Li, X. (2012). Influence of dielectric and machining parameters on the process performance for electric discharge milling of SiC ceramic.* Int. J. Adv. Manufact. Technol.*** 59** (1),127–136.

[46] Sakai, K., Kobayashi, Y., Saito, T. & Isogai, A. Partitioned airs at microscale and nanoscale: thermal diffusivity in ultrahigh porosity solids of nanocellulose. *Sci. Rep.* **6**, 20434 (2016). 26830144 10.1038/srep20434 PMC4735846

[47] Sun, J. et al. High-temperature ablation resistance prediction of ceramic coatings using machine learning. *J. Am. Ceram. Soc.* **108** (1), e20136. 10.1111/jace.20136 (2025).

[48] Su, L., Wu, S., Fu, G., Zhu, W., Zhang, X.,… Liang, B. (2024). Creep characterisation and microstructural analysis of municipal solid waste incineration fly ash geopolymer backfill.* Sc. Rep.*** 14** (1), 29828. 10.1038/s41598-024-81426-7 PMC11608240 39616225

[49] Xiao, Y., Cheng, D., Li, G., Yin, R., Li, P., Gao, Z. (2025). Preparation of MgO ceramics by low temperature sintering with MgF2 and Al2O3 as sintering additives.*J. Electroceramics*. 10.1007/s10832-025-00391-3.

[50] Liu, B., Sun, J., Guo, L., Shi, H., Feng, G., Feldmann, L.,… Li, H. (2025). Materials design of silicon based ceramic coatings for high temperature oxidation protection.*Materials Sci. Eng. R: Rep.*,** 163**, 100936.

[51] Meng, X. et al. Effect of porosity and pore density of copper foam on thermal performance of the paraffin-copper foam composite Phase-Change material. *Case Stud. Therm. Eng.* **22**, 100742. 10.1016/j.csite.2020.100742 (2020).

[52] Holman, J. P. (ed) (etc.) Heat Transfer (8th edition), (McGraw-Hill Book Company,1997). New York, 56–59

[53] Ruuska, T., Vinha, J. & Kivioja, H. Measuring thermal conductivity and specific heat capacity values of inhomogeneous materials with a heat flow meter apparatus. *J. Building Eng.* **9**, 135–141. 10.1016/j.jobe.2016.11.011 (2017).

[54] Hamzawy, E. M. A., El-Kheshen, A. A. & Zawrah, M. F. Densification and properties of glass/cordierite composites. *Ceram. Int.* **31**, 383–389. 10.1016/j.ceramint.2004.06.003 (2005).

[55] Hamzawy, E. M. A., Abd El-Shakour, Z. A., El-Kheshen, A. A., El-Bassyouni, G. T. & Zawrah, M. F. Fabrication of Sr-feldspar/cordierite and Sr-feldspar/Sr-osumilite composites through sintering of Mg-Sr-cordierite and borosilicate glass for electronic applications. *Ceram. Int.* **50** (10), 16852–16857 (2024).

